# Correction: Fluorescent sensors reporting the activity of ammonium transceptors in live cells

**DOI:** 10.7554/eLife.06986

**Published:** 2015-02-18

**Authors:** Roberto De Michele, Cindy Ast, Dominique Loqué, Cheng-Hsun Ho, Susana LA Andrade, Viviane Lanquar, Guido Grossmann, Sören Gehne, Michael U Kumke, Wolf B Frommer

De Michele R, Ast C, Loqué D, Ho CH, Andrade SL, Lanquar V, Grossmann G, Gehne S, Kumke MU, Frommer WB. 2013. Fluorescent sensors reporting the activity of ammonium transceptors in live cells. *eLife* **2**:e00800. doi: 10.7554/eLife.00800. Published 2 July 2013

In the published article, Amtrac kinetics derived from the same dataset were intentionally shown for comparison in three different figures (Figures 4C, 5D, and 8B). For clarification, a corresponding statement has now been added to the respective legends. The Amtrac fluorescence response kinetics were repeated multiple times independently with similar results.

Figure 2 of the published article contained an error: the graph showed two sensors labelled #16 (see below).

**Figure 1:**
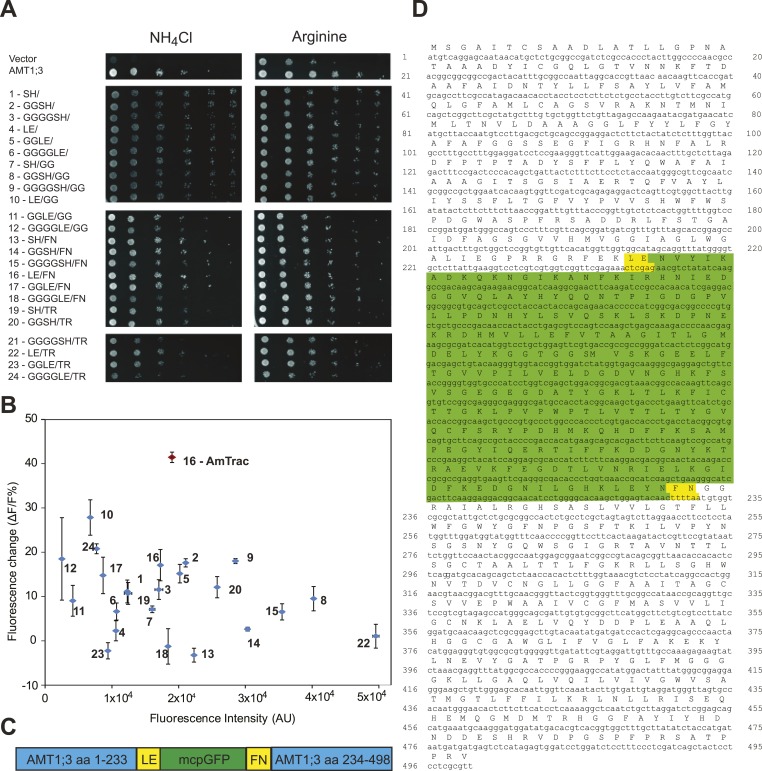

The upper of the two was correctly labelled #16 (in red). The lower should have been labelled #21 (see below).

**Figure 2:**
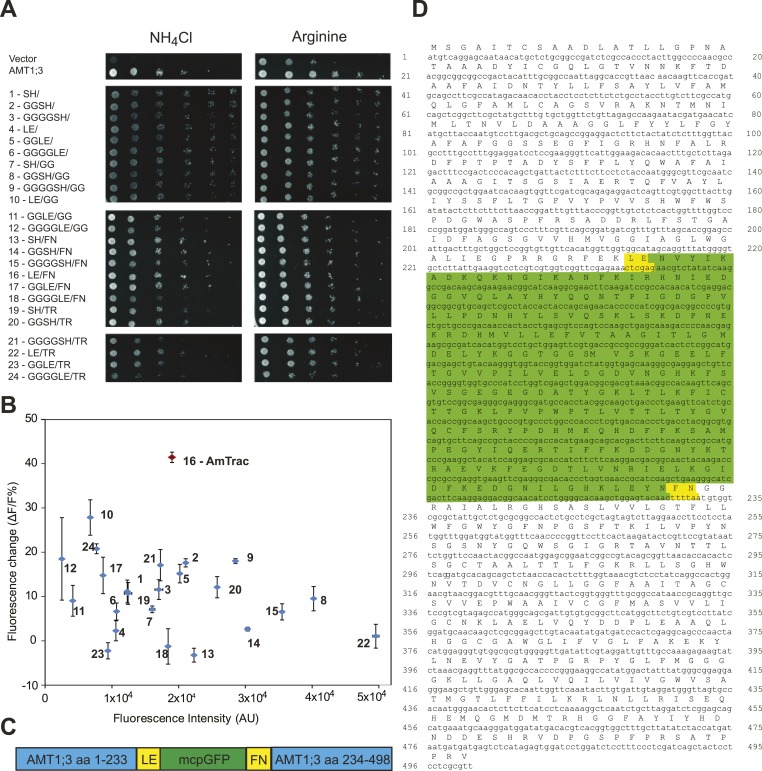

In addition, in Figure 7D, the 11th TMH of *Archaeoglobus fulgidus* was highlighted in grey, but this was not explained in the legend.

The article has been corrected accordingly.

